# Synthesis and Evaluation of Novel Pyrroles and Pyrrolopyrimidines as Anti-Hyperglycemic Agents

**DOI:** 10.1155/2014/249780

**Published:** 2014-06-26

**Authors:** M. S. Mohamed, S. A. Ali, D. H. A. Abdelaziz, Samar S. Fathallah

**Affiliations:** ^1^Pharmaceutical Organic Chemistry Department, Faculty of Pharmacy, Helwan University, Ein-Helwan, Helwan, Cairo 11795, Egypt; ^2^Biochemistry and Molecular Biology Department, Faculty of Pharmacy, Helwan University, Ein-Helwan, Helwan, Cairo 11795, Egypt

## Abstract

A series of pyrrole and pyrrolopyrimidine derivatives were examined for their *in vivo* antihyperglycemic activity. Compounds **Ia**–**c**,**e**, and **IVg** showed promising antihyperglycemic activity equivalent to a well-known standard antihyperglycemic drug, Glimepiride (Amaryl, 4 mg/kg). In this paper, we examine and discuss the structure-activity relationships and antihyperglycemic activity of these compounds.

## 1. Introduction

**Figure 1:**
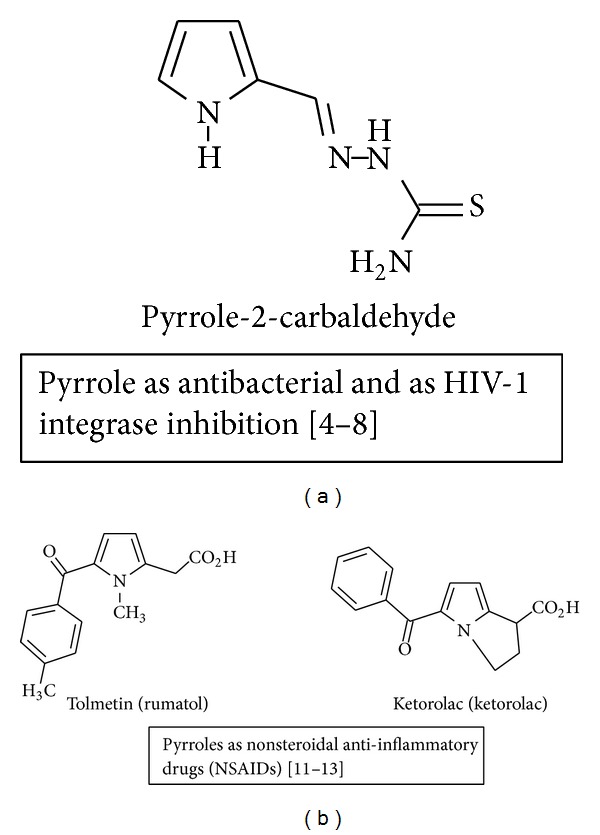
Pyrrole as valuable leads in the drug discovery field.

**Figure 2:**
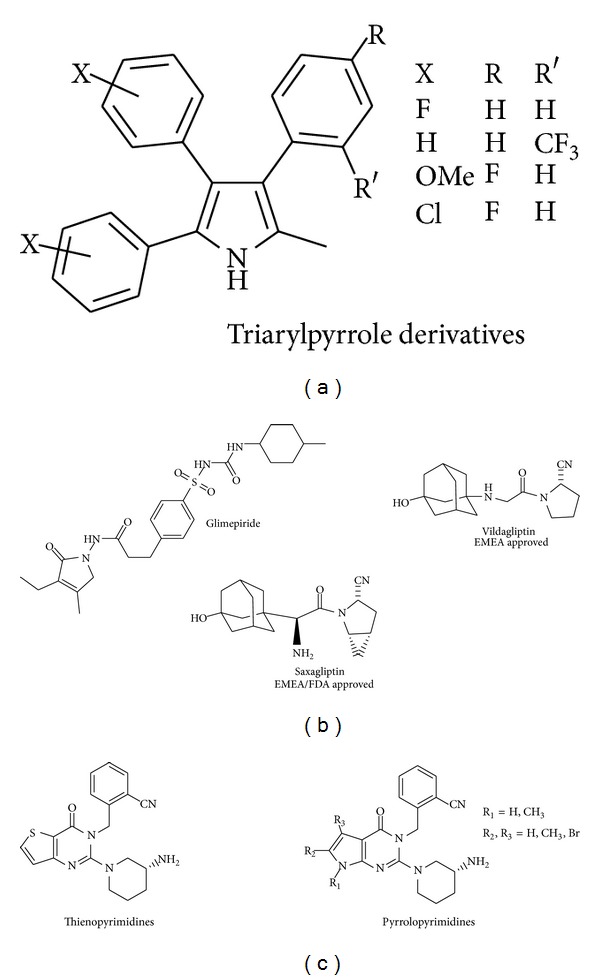
(a) Pyrroles as antihyperglycemic agents [19, 20], (b) Amaryl, standard antihyperglycemic drug [21, 22], and approved DPP-IV inhibitors [23–25] as type 2 diabetes medications containing a pyrrole moiety. (c) Thieno and Pyrrolo-pyrimidines as DPP-IV inhibitors.

For several decades, interest in pyrrole derivatives increases due to their pharmaceutical importance [1–3], such as antimicrobial [4–8], antiviral [9, 10], anti-inflammatory [11–13], analgesic [14], antitumor [15, 16], antihyperlipidemic [17], anticonvulsant [18], and antihyperglycemic agents [19, 20], as shown in Figures 1 and 2(a).

Likewise, the key roles played by purines and pyrimidines in cellular processes have made them valuable lead for drug discovery; among these, pyrrolo[3,2-*d*]pyrimidines, a class of 7-deazapurine analogs, exhibit interesting biological activity in part due to their resemblance to pyrimidines and purines. These huge therapeutic applications have motivated new efforts in the search for novel derivatives with improved biological activity and diverse applications in the pharmaceutical industry [1–4, 19, 20].

Diabetes mellitus (DM) is a severe metabolic disorder that has a significant impact on the health and quality of patients' life. Treatment of diabetic patients has been focused on dietary management and oral antidiabetics, among these: sulfonylureas, metformin, acarbose, and others. However, some of the currently used antihyperglycemic have several adverse side effects like hepatotoxicity, weight gain, and hypoglycemia.

This situation emphasized the need to develop novel antihyperglycemic agents [21]. Glimepiride (Amaryl) is a sulfonylurea containing a pyrrole group, acting as antihyperglycemic drug [22]. It is sometimes classified either as the first third-generation sulfonylurea or as second-generation. Glimepiride is indicated to treat type 2 diabetes mellitus; its mode of action is to increase insulin production by the pancreas, as shown in Figure 2(b).

Recently, dipeptidyl peptidase IV (DPP-IV) inhibitors [23–25] have been shown to be effective and safe compounds that control blood glucose. Improvement of the inhibitory activity and chemical stability of a series of substituted piperidinyl glycine 2-cyano-4,5-methano pyrroline (DPP-IV) inhibitors was, respectively, achieved by the introduction of pyrroline moiety at the 4 position and 1 position of the piperidinyl glycine, leading to a series of potent and stable DPP-IV inhibitors [25]. Two important DPP-IV inhibitors, having a pyrrole and fused pyrrole, vildaglipin, and saxagliptin [24, 25], are on the market in many countries, as shown in Figure 2(b).

A highly potent DPP-IV inhibitor thienopyrimidine was also reported [24]. While trying to maintain consistency of* in vitro* and* in vivo* biological activity, a simple scaffold replacement of thienopyrimidine with pyrrolopyrimidine lead to significantly improved metabolic stability [22–24], as shown in Figure 2(c).

**Scheme 1:**
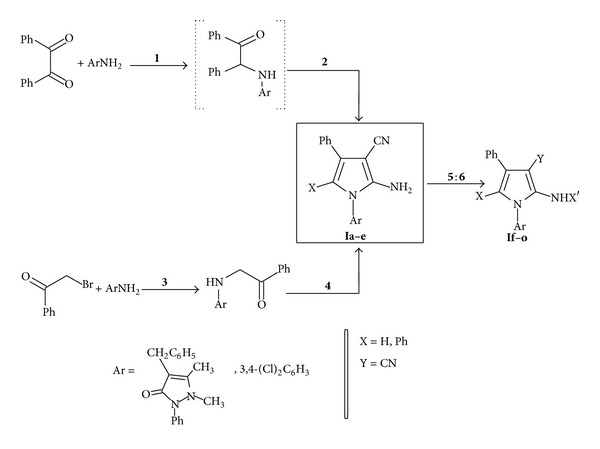
Synthetic pathways for compounds** Ia–o**: reagents and conditions: (**1**) pyridine/benzene; (**2**) CH_2_(CN)_2_; (**3**) NaHCO_3_/EtOH; (**4**) CH_2_(CN)_2_/NaOEt; (**5**) TEOF; or (**6**) Ac_2_O.

**Scheme 2:**
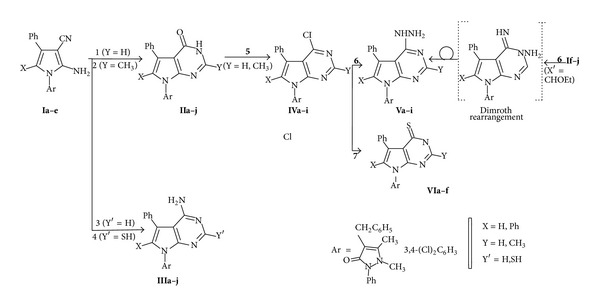
Synthetic pathways for compounds** I–VI**: reagents and conditions: (**1**) HCO_2_H; (**2**) AcOH/HCl; (**3**) HCONH_2_; (**4**) NH_2_CSNH_2_; (**5**) POCl_3_; (**6**) N_2_H_4*·*_H_2_O; and (**7**) NH_2_CSNH_2_.

**Scheme 3:**
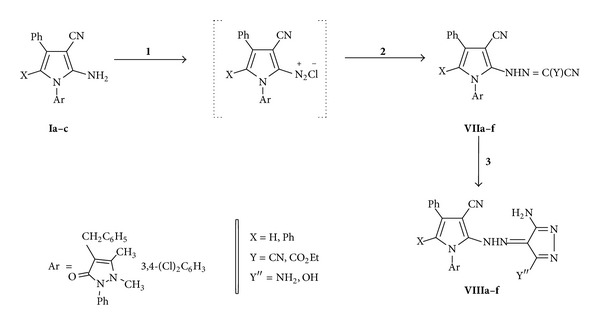
Synthetic pathways for compounds** VII–VIII**: reagents and conditions: (**1**) NaNO_2_/HCl/Stirring (75 min); (**2**) NCCH_2_Y/CH_3_CO_2_NH_4_/EtOH; and (**3**) N_2_H_4_·H_2_O.

Motivated by the importance of this system and in continuation of our research efforts [26–30], we try to highlight aspects reported on the chemistry of some newly synthesized pyrrole and pyrrolopyrimidine derivatives and evaluate them for the antihyperglycemic activities. The synthetic pathways adopted for the synthesis of these compounds are registered in Schemes 1–3.

## 2. Materials and Methods

### 2.1. Chemistry

All melting points were uncorrected and measured using Electrothermal IA 9100 apparatus (Shimadzu, Japan). IR spectra were recorded as potassium bromide pellets on a Perkin-Elmer 1650 spectrophotometer (USA), Faculty of Science, Cairo University, Cairo, Egypt. ^1^H NMR and ^13^CNMR spectra were performed on JOEL NMR FXQ-300 MHz and JOELNMR FXQ-500 MHz spand chemical shifts were expressed as ppm against TMS as internal reference (Faculty of Science, Cairo University, Cairo, Egypt). Mass spectra were recorded at 70 eV EI Ms-QP 1000 EX (Shimadzu, Japan), Faculty of Science, Cairo University, Cairo, Egypt. Microanalyses were operated using Vario, Elementar apparatus (Shimadzu, Japan), Organic Microanalysis Unit, Faculty of Science, Cairo University, Cairo, Egypt. Column chromatography was performed on (Merck) silica gel 60 (particle size 0.06–0.20 mm). Compounds** Ia–c**,** f–h**,** k–m**,** IIa–c**,** f–h**,** IIIa–c**,** f–h**,** IVa–c**,** Va–c**,** VIa–c**,** VIIa–c**,and** VIIIa–c** were prepared as reported in the literature [26–31]. All new compounds yielded spectral data consistent with the proposed structure and microanalysis within ± 0.4% of the theoretical values.

#### 2.1.1. 2-Amino-1-(3,4-dichlorophenyl)-4,5-diphenyl-*1H*-pyrrole-3-carbonitrile** Id** (Scheme 1)

A mixture of benzoin (2 g, 0.01 mol), 3,4-dichlorophenyl amine (1.6 g, 0.01 mol) in dry benzene (50 mL), was kept at 80°C for 9 h. The reaction mixture was cooled; then malononitrile (0.66 mg, 0.01 mol) was added, followed by catalytic amount of pyridine (2 mL) portion wise and left to reflex till solid formed. The solvent was evaporated under reduced pressure and the residue was recrystallized from methanol to give** Id.** Yield: 45%; M.P. 118–122°C; ^1^H NMR (DMSO-*d*_6_, 300 MHz) *δ* (ppm): 5.21 (br.s, 2H, NH_2_, D_2_O exchangeable), 7.0–7.8 (m, 13H, Ar-H); ^13^C NMR (DMSO-*d*_6_): *δ* 114.33, 118.24, 119.37, 125.8, 126.18, 127.80, 128.45, 129.84, 130.29, 132.16, 132.70, 133.62, 134.96, 142.05 ppm; IR (KBr) *υ* (cm^−1^): 3410, 3370 (NH_2_), 2220 (C*≡*N); MS (EI)* m/z*: 403 (M^+^, 14%), 405 (M^+^+ 2, 8.75%), 407 (M^+^+ 4, 1.1%); Anal. Calcd for C_23_H_15_Cl_2_N_3_ (403.06): C, 68.33; H, 3.74; Cl, 17.54; N, 10.39%. Found: C, 68.55; H, 3.92; Cl, 17.23; N, 10.72%.

#### 2.1.2. 2-Amino-1-(1,5-dimethyl-3-oxo-2-phenyl-2,3-dihydro-*1H* -pyrazol-4-yl)-4-phenyl-*1H* -pyrrole-3-carbonitrile** Ie** (Scheme 1)

1,5-Dimethyl-4-(2-oxo-2-phenylethylamino)-2-phenyl-*1H*-pyrazol-3(*2H*)-one [27–30](3.22 g, 0.01 mol) was dissolved in dry ethanol (20 mL); then malononitrile (0.66 g, 0.01 mol) was added, followed by sodium ethoxide (0.01 mol) portion wise, and left to reflux till solid formed. The solvent was evaporated under reduced pressure and the residue was recrystallized from methanol to give** Ie**.Yield: 66%; M.P. 163–166°C;^ 1^H NMR (DMSO-*d*_6_, 300 MHz) *δ* (ppm): 2.43 (s, 3H, CH_3_), 3.12 (s, 3H, N-CH_3_), 6.13 (br.s, 2H, NH_2_, D_2_O exchangeable), 6.8–7.8 (m, 10H, Ar-H and 1H, C_6_-H); IR (KBr) *υ* (cm^−1^): 3410, 3350 (NH_2_), 2210 (C*≡*N), 1703 (C=O); MS (EI)* m/z*: 369 (M^+^, 23%), 370 (M^+^+ 1, 6.1%); Anal. Calcd for C_22_H_19_N_5_O (369.16): C, 71.53; H, 5.18; N, 18.96; O, 4.33%. Found: C, 71.55; H, 5.26; N, 18.70; O, 3.05%.

#### 2.1.3. 2-Ethoxymethylenamino-1,5-disubstituted-4-phenyl-*1H* -pyrrole-3-carbonitriles** Ii,j** (Scheme 1)

Compound** I**,** d**,** or e** (0.01 mol) in triethyl orthoformate (20 mL) was refluxed for 9 h. The solvent was removed under reduced pressure and the residue was recrystallized from methanol/water to give the target compounds** Ii**,** j**.

#### 2.1.4. Ethyl N-3-Cyano-1-(3,4-dichlorophenyl)-4,5-diphenyl-*1H* -pyrrol-2-ylformimidate** Ii**

Yield: 60%; M.P. 120–122°C; ^1^H NMR (DMSO-*d*_6_, 300 MHz) *δ* (ppm): 1.30 (t, 3H,* J* = 7.1 Hz, CH_3_), 4.2 (q, 2H,* J *= 7.1 Hz, OCH_2_), 6.9–7.8 (m, 14H, Ar-H and N=CH); IR (KBr) *υ* (cm^−1^): 3070, 2900(CH), 2310(CN), 1620 (C=C), 1560 (C=N); MS (EI)* m/z:* 460 (M^+^, 13.5%), 462 (M^+^+ 2, 8.5%), 464 (M^+^+ 4, 2.71%); Anal. Calcd for C_26_H_19_Cl_2_N_3_O (460.35): C, 67.83; H, 4.16; Cl, 15.40; N, 9.13; O, 3.48%, Found: C, 68.03; H, 4.11; Cl, 15.63; N, 8.91; O, 3.69%.

#### 2.1.5. Ethyl N-3-Cyano-1-(1,5-dimethyl-3-oxo-2-phenyl-2,3-dihydro-*1H* -pyrazol-4-yl)-4-phenyl-*1H* -pyrrol-2-ylacetimidate** Ij**

Yield: 56%; M.P. 120–122°C; ^1^H NMR (DMSO-*d*_6_, 300 MHz) *δ* (ppm): 1.20 (t, 3H,* J* = 7.2 Hz, CH_3_), 2.41 (s, 3H, CH_3_), 3.1 (s, 3H, N-CH_3_), 4.01 (q, 2H,* J *= 7.1 Hz, OCH_2_), 6.9–7.8 (m, 12H, Ar-H, C_6_-H and N=CH); IR (KBr) *υ* (cm^−1^): 3030, 2910(CH), 2240(CN), 1610 (C=C), 1570 (C=N); MS (EI)* m/z:* 425 (M^+^, 6.5%), 426 (M^+^+ 1, 1.81%); Anal. Calcd for C_26_H_23_N_5_O_2_ (425.19): C, 70.57; H, 5.45; N, 16.46; O, 7.52%. Found: C, 70.21; H, 5.17; N, 16.12; O, 7.18%.

#### 2.1.6. N-(4-Phenyl-1,3,5-trisubstituted-*1H* -pyrrol-2-yl)-acetamides** I n,o** (Scheme 1)

Compound** I**,** d**, or** e** (0.01 mol) in acetic anhydride (30 mL) was refluxed for 2 h, cooled, poured onto ice water, neutralized with ammonia to give a precipitate which was filtered off, dried, and recrystallized from methanol to give the target compounds** In**,** o**.

#### 2.1.7. N-(3-Cyano-1-(3,4-dichlorophenyl)-4,5-diphenyl-*1H* -pyrrol-2-yl) acetamide** In**

Yield: 66%; M.P. 135–138°C; ^1^H NMR (DMSO-*d*_6_, 300 MHz) *δ* (ppm): 2.23 (s, 3H, CO-CH_3_), 7.0–7.8 (m, 13H, Ar-H), 9.5 (s, 1H, NH, D_2_O exchangeable); IR (KBr) *υ* (cm^−1^): 3430 (NH), 2330 (C*≡*N), 1710 (C=O); MS (EI)* m/z:* 445 (M^+^, 13.1%), 447 (M^+^ + 2, 7.9%), 449 (M^+^ + 4, 0.91%); Anal. Calcd for C_25_H_17_Cl_2_N_3_O (445.07): C, 67.28; H, 3.84; Cl, 15.89; N, 9.41; O, 3.58%. Found: C, 67.47; H, 4.06; Cl, 16.22; N, 9.57; O, 3.66%.

#### 2.1.8. N-(3-Cyano-1-(1,5-dimethyl-3-oxo-2-phenyl-2,3-dihydro-*1H* -pyrazol-4-yl)-4-phenyl-*1H* -pyrrol-2-yl)acetamide** Io**

Yield: 72%; M.P. 135–138°C; ^1^H NMR (DMSO-*d*_6_, 300 MHz) *δ* (ppm): 2.23 (s, 3H, CO-CH_3_), 2.43 (s, 3H, CH_3_), 3.12 (s, 3H, N-CH_3_), 7.0–7.8 (m, 11H, Ar-H and C_6_-H), 9.4 (s, 1H, NH, D_2_O exchangeable); IR (KBr) *υ* (cm^−1^): 3350 (NH), 2310 (C*≡*N), 1715, 1705 (C=O); MS (EI)* m/z:* 411 (M^+^, 15.4%), 412 (M^+^+ 1, 3.73%); Anal. Calcd for C_24_H_21_N_5_O_2_ (411.17): C, 70.06; H, 5.14; N, 17.02; O, 7.78%. Found: C, 70.37; H, 5.45; N, 17.34; O, 7.95%.

#### 2.1.9. 7-(2,4-Dimethyl-5-oxo-1-phenyl-2,5-dihydro-*1H* -pyrazol-3-yl)-5,6-diphenyl -3H-pyrrolo[2,3-d]pyrimidin-4(7H)-one** IId,e** (Scheme 2)

Compound** Id** or** e** (0.01 mol) in formic acid (20 mL, 85%) was refluxed for 12 h. The reaction mixture was cooled, poured onto ice/water to give a precipitate which was filtered, dried, and recrystallized from ethanol to give the target compounds** IId**,** e**. 

7*-(3,4-Dichlorophenyl)-5,6-diphenyl-3H-pyrrolo[2,3-d]pyrimidin-4(7H)-one* ***IId***. Yield: 56%; M.P. 172–176°C; ^1^H NMR (DMSO-*d*_6_, 300 MHz) *δ* (ppm): 6.9–7.8 (m, 13H, Ar-H), 8.4 (s, 1H, C_2_-H), 12.40 (s, 1H, NH, D_2_O exchangeable): ^13^C NMR (DMSO-*d*_6_): *δ*118.24, 119.37, 125.8, 126.18, 127.80, 128.45, 129.84, 130.29, 132.16, 132.70, 133.62, 134.96, 138.25, 142.05, 146.2, 163.15 ppm; IR (KBr) *υ* (cm^−1^): 3410, 3350 (NH_2_), 1720, 1705 (C=O), 1550(C=N); MS (EI)* m/z:* 432 (M^+^, 21.5%), 434 (M^+^+ 2, 13.9%), 436 (M^+^+ 4, 4.2%); Anal. Calcd for C_24_H_15_Cl_2_N_3_O (432.30): C, 66.68; H, 3.50; Cl, 16.40; N, 9.72; O, 3.70%. Found: C, 66.32; H, 3.18; Cl, 16.21; N, 9.50; O, 3.46%.

7*-(1,5-Dimethyl-3-oxo-2-phenyl-2,3-dihydro-1H-pyrazol-4-yl)-5-phenyl-3H-pyrrolo[2,3-d]pyrimidin-4(7H)-one* ***IIe***. Yield: 65%; M.P. 205–208°C; ^1^H NMR (DMSO-*d*_6_, 300 MHz) *δ* (ppm): 2.41 (s, 3H, CH_3_), 3.21 (s, 3H, N-CH_3_), 6.9–7.8 (m, 11H, Ar-H and C_6_-H), 8.32 (s, 1H, C_2_-H), 12.32 (s, 1H, NH, D_2_O exchangeable); IR (KBr) *υ* (cm^−1^): 3410, 3320 (NH_2_), 1720, 1680 (C=O), 1560(C=N) and disappearance of the CN group; MS (EI)* m/z:* 379 (M^+^, 23.5%), 380 (M^+^+ 1, 7.1%); Anal. Calcd for C_23_H_19_N_5_O_2_ (379.43): C, 69.51; H, 4.82; N, 17.62; O, 8.05%. Found: C, 69.89; H, 5.16; N, 17.97; O, 8.41%.

#### 2.1.10. 7-Disubstituted-2-methyl-5-phenyl-7H-pyrrolo[2,3-d]pyrimidin-4(3H)-one** IIi, j** (Scheme 2)

Compound** Id** or** e** (0.01 mol) in acetic acid/HCl (3 : 1) (30 mL) was refluxed for 12 h. The reaction mixture was cooled, poured onto ice/water, neutralized with ammonia to give a precipitate which was filtered, dried, and recrystallized from methanol to give the target compounds** II i**,** j**.

7*-(3,4-Dichlorophenyl)-2-methyl-5,6-diphenyl-3H-pyrrolo[2,3-d]pyrimidin-4(7H)-one* ***II i***. Yield: 67%; M.P. 200–202°C; ^1^H NMR (DMSO-*d*_6_, 300 MHz) *δ* (ppm): 2.35 (s, 3H, C_2_-CH_3_), 6.9–7.8 (m, 13H, Ar-H), 12.40 (s, 1H, NH, D_2_O exchangeable); IR (KBr) *υ* (cm^−1^): 3430, 3330 (NH_2_), 1710 (C=O), 1570 (C=N); MS (EI)* m/z:* 445 (M^+^, 26.2%), 447 (M^+^+ 2, 15.3%), 449 (M^+^+ 4, 1.04%); Anal. Calcd for C_25_H_17_Cl_2_N_3_O (445.07): C, 67.28; H, 3.84; Cl, 15.89; N, 9.41; O, 3.58%. Found: C, 67.45; H, 4.11; Cl, 16.22; N, 9.74; O, 3.82%.

7*-(1,5-Dimethyl-3-oxo-2-phenyl-2,3-dihydro-1H-pyrazol-4-yl)-2-methyl-5-phenyl-3H-pyrrolo[2,3-d]pyrimidin-4(7H)-one* ***II j***. Yield: 67%; M.P. 225–227°C; ^1^H NMR (DMSO-*d*_6_, 300 MHz) *δ* (ppm): 2.32 (s, 3H, C_2_-CH_3_), 2.41 (s, 3H, CH_3_), 3.22 (s, 3H, N-CH_3_), 7–7.8 (m, 11H, Ar-H and C_6_-H), 12.36 (s, 1H, NH, D_2_O exchangeable); IR (KBr) *υ* (cm^−1^): 3430, 3330 (NH_2_), 1730, 1700 (C=O), 1560 (C=N); MS (EI)* m/z:* 487 (M^+^, 45%), 488 (M^+^+ 1, 15.2%); Anal. Calcd for C_24_H_21_N_5_O_2_ (411.46): C, 70.06; H, 5.14; N, 17.02; O, 7.78%. Found: C, 70.24; H, 5.32; N, 17.31; O, 7.96%.

#### 2.1.11. 6,7-Disubstituted-5-phenyl-7H-pyrrolo[2,3-d]pyrimidin-4-ylamines** IIId, e** (Scheme 2)

A suspension of the appropriate aminopyrrole** I**,** d**, or** e** (0.01 mol) and formamide (30 mL, 0.066 mol) were heated under reflux for 9 h, cooled, poured onto ice/water to give precipitates which were filtered off, dried, and recrystallized from ethanol to give the target compounds** III d**,** e**.

*7-(3,4-Dichlorophenyl)-5,6-diphenyl-7H-pyrrolo[2,3-d]pyrimidin-4-amine* ***III d***. Yield: 63%; M.P. 115–118°C; ^1^H NMR (DMSO-*d*_6_, 300 MHz) *δ* (ppm): 5.4 (brs, 2H, NH_2_, D_2_O exchangeable), 7.1–7.7 (m, 13H, Ar-H), 8.2 (s, 1H, C_2_-H); IR (KBr) *υ* (cm^−1^): 3440, 3350 (NH_2_), 1570 (C=N), 1610 (C=C); MS (EI)* m/z:* 430 (M^+^, 31.2%), 432 (M^+^+ 2, 17.6%), 434 (M^+^+ 4, 0.98%); Anal. Calcd for C_24_H_16_Cl_2_N_4_ (430.08): C, 66.83; H, 3.74; Cl, 16.44; N, 12.99%. Found: C, 67.07; H, 5.11; Cl, 16.81; N, 13.36%.

*4-(4-Amino-5-phenyl-7H-pyrrolo[2,3-d]pyrimidin-7-yl)-1,5-dimethyl-2-phenyl-1H-pyrazol-3(2H)-one* ***III e***. Yield: 69%; M.P. 152–155°C; ^1^H NMR (DMSO-*d*_6_, 300 MHz) *δ* (ppm): 2.41 (s, 3H, CH_3_), 3.14 (s, 3H, N-CH_3_), 5.8 (brs, 2H, NH_2_, D_2_O exchangeable), 7.1–7.8 (m, 11H, Ar-H, and C_6_-H), 8.3 (s, 1H, C_2_-H); IR (KBr) *υ* (cm^−1^): 3430, 3330 (NH, NH_2_), 1705 (C=O), 1600 (C=C), 1540 (C=N); MS (EI)* m/z:* 396 (M^+^, 13.7%), 397 (M^+^+ 1, 1.91%); Anal. Calcd for C_23_H_20_N_6_O (396.44): C, 69.68; H, 5.08; N, 21.20; O, 4.04%. Found: C, 69.42; H, 4.92; N, 20.89; O, 4.32%.

#### 2.1.12. 5-(5,6-Diphenyl-4-thioxo-3H-pyrrolo[2,3-d]pyrimidin-7(4H)-yl)-1,4-dimethyl-2-phenyl-*1H* -pyrazol-3(2H)-one** IIIi, j** (Scheme 2)

Compound** I**,** d**, or** e** (0.01 mol) and thiourea (1.2 g, 0.02 mol) were refluxed in dry ethanol (20 mL) for 12 h. The reaction mixture was evaporated under reduced pressure and the residues were recrystallized from methanol to give the target compounds** III i**,** j**.

*4-Amino-7-(3,4-dichlorophenyl)-5,6-diphenyl-1H-pyrrolo[2,3-d] pyrimidine-2(7H)-thione* ***IIIi***. Yield: 70%; M.P. 90–95°C; ^1^H NMR (DMSO-*d*_6_, 300 MHz) *δ* (ppm): 6.62 (s, 2H, NH_2_, D_2_O exchangeable), 7.1–7.9 (m, 13H, Ar-H), 9.22 (s, 1H, NH, D_2_O exchangeable); IR (KBr) *υ* (cm^−1^): 3440, 3340 (NH, NH_2_), 1610 (NH–C=S); MS (EI)* m/z:* 463 (M^+^, 22.36%), 465 (M^+^+ 2, 14.23%), 467 (M^+^ + 4, 4.54%); Anal. Calcd for C_24_H_16_Cl_2_N_4_S (463.38): C, 62.21; H, 3.48; Cl, 15.30; N, 12.09; S, 6.92%. Found: C, 62.45; H, 3.62; Cl, 15.49; N, 12.41; S, 7.21%.

*4-(4-Amino-5-phenyl-2-thioxo-1H-pyrrolo[2,3-d]pyrimidin-7(2H)-yl)-1,5-dimethyl-2-phenyl-1H-pyrazol-3(2H)-one* ***IIIj***. Yield: 74%; M.P. 172–174°C; ^1^H NMR (DMSO-*d*_6_, 300 MHz) *δ* (ppm): 2.34 (s, 3H, CH_3_), 3.11 (s, 3H, N-CH_3_), 6.51 (brs, 2H, NH_2_, D_2_O exchangeable), 7.1–7.8 (m, 11H, Ar-H, and C_6_-H), 8.3 (s, 1H, C_2_-H), 8.92 (s, 1H, NH, D_2_O exchangeable); IR (KBr) *υ* (cm^−1^): 3440, 3370 (NH, NH_2_), 1610 (C=C), 1600 (NH–C=S); MS (EI)* m/z:* 428 (M^+^, 14.8%), 429 (M^+^ + 1, 2.31%); Anal. Calcd for C_23_H_20_N_6_OS (428.51): C, 64.47; H, 4.70; N, 19.61; O, 3.73; S, 7.48%. Found: C, 64.78; H, 4.97; N, 19.92; O, 3.91; S, 7.62%.

#### 2.1.13. General Procedure for the Preparation of 4-Chloropyrrolopyrimidines** IVd–j** (Scheme 2)

The appropriate compound** II** (0.01 mol) was refluxed in phosphorus oxychloride (30 mL) for 12 h. The solution was cooled and poured with stirring onto ice/water and the formed precipitated was filtered, washed several times with water, dried, and recrystallized from ethanol to give the target compounds** IVd–j**.

*4-Chloro-7-(3,4-dichlorophenyl)-5,6-diphenyl-7H-pyrrolo[2,3-d]pyrimidine* ***IVd***. Yield: 76%; M.P. 124–128°C; ^1^H NMR (DMSO-*d*_6_, 300 MHz) *δ* (ppm): 6.9–7.8 (m, 13H, Ar-H), 8.6 (s, 1H, C_2_-H); ^13^C NMR (DMSO-*d*_6_): *δ*117.5, 118.8, 126.18, 127.80, 128.45, 129.84, 130.21, 132.46, 132.64, 133.76, 134.84, 138.27, 141.05, 150.2, 151.24, 153.8 ppm; IR (KBr) *υ* (cm^−1^): 3080, 2840(CH), 1612 (C=C), 1580 (C=N); MS (EI)* m/z:* 449 (M^+^, 29.98%), 451 (M^+^ + 2, 23.6%), 453 (M^+^ + 4, 6.9%), 455 (M^+^ + 6, 0.66%); Anal. Calcd for C_24_H_14_Cl_3_N_3_ (449.03): C, 63.95; H, 3.13; Cl, 23.60; N, 9.32%. Found: C, 64.23; H, 3.42; Cl, 23.91; N, 9.49%.

*4-(4-Chloro-5-phenyl-7H-pyrrolo[2,3-d]pyrimidin-7-yl)-1,5-dimethyl-2-phenyl-1H-pyrazol-3(2H)-one* ***IVe***. Yield: 76%; M.P. 125–130°C; ^1^H NMR (DMSO-*d*_6_, 300 MHz) *δ* (ppm): 2.43 (s, 3H, CH_3_), 3.12 (s, 3H, N-CH_3_), 6.9–7.8 (m, 11H, Ar-H and C_6_-H), 8.5 (s, 1H, C_2_-H); IR (KBr) *υ* (cm^−1^): 3080, 2840 (CH), 1730 (C=O), 1612 (C=C), 1580 (C=N); MS (EI)* m/z:* 415 (M^+^, 20%), 417 (M^+^+ 2, 5.5%); Anal. Calcd for C_23_H_18_ClN_5_O (415.87): C, 66.43; H, 4.36; Cl, 8.52; N, 16.84; O, 3.85%. Found: C, 66.67; H, 4.71; Cl, 8.68; N, 16.94; O, 3.99%.

*7-Benzyl-4-chloro-2-methyl-5,6-diphenyl-7H-pyrrolo[2,3-d]pyrimidine* ***IVf***. Yield: 46%; M.P. 120–124°C; ^1^H NMR (DMSO-*d*_6_, 300 MHz) *δ* (ppm): 2.32 (s, 3H, CH_3_), 5.12 (s, 2H, Ph-CH_2_), 6.9–7.8 (m, 15H, Ar-H and C_6_-H); IR (KBr) *υ* (cm^−1^): 3080, 2840 (CH), 1610 (C=C), 1560 (C=N); MS (EI)* m/z:* 409 (M^+^, 14%), 411 (M^+^ + 2, 4.5%); Anal. Calcd for C_26_H_20_ClN_3_ (409.91): C, 76.18; H, 4.92; Cl, 8.65; N, 10.25%. Found: C, 76.47; H, 5.23; Cl, 8.96; N, 10.59%.

*4-(4-Chloro-2-methyl-5,6-diphenyl-7H-pyrrolo[2,3-d]pyrimidin-7-yl)-1,5-dimethyl-2-phenyl-1H-pyrazol-3(2H)-one* ***IVg***. Yield: 42%; M.P. 158–160°C; ^1^H NMR (DMSO-*d*_6_, 300 MHz) *δ* (ppm): 2.30–2.44 (s, 6H,2∗CH_3_), 3.13 (s, 3H, N-CH_3_), 6.9–7.8 (m, 15H, Ar-H); IR (KBr) *υ* (cm^−1^): 3080, 2840 (CH), 1710 (C=O), 1605 (C=C), 1577 (C=N); MS (EI)* m/z:* 505 (M^+^, 13%), 507 (M^+^ + 2, 2.5%); Anal. Calcd for C_30_H_24_ClN_5_O (505.17): C, 71.21; H, 4.78; Cl, 7.01; N, 13.84; O, 3.16%. Found: C, 71.02; H, 4.97; Cl, 7.23; N, 14.06; O, 3.45%.

*4-Chloro-7-(3,4-dichlorophenyl)-2-methyl-5,6-diphenyl-7H-pyrrolo[2,3-d]pyrimidine* ***IVh***. Yield: 45%; M.P. 108–110°C; ^1^H NMR (DMSO-*d*_6_, 300 MHz) *δ* (ppm): 2.36 (s, 3H, CH_3_), 7.2–7.8 (m, 13H, Ar-H); IR (KBr) *υ* (cm^−1^): 3080, 2840 (CH), 1600 (C=C), 1565 (C=N); MS (EI)* m/z:* 464 (M^+^, 11%), 466 (M^+^ + 2, 6.5%), 468 (M^+^ + 4, 2.3%), 394 (M^+^ + 6, 0.88%); Anal. Calcd for C_25_H_16_Cl_3_N_3_ (464.77): C, 64.61; H, 3.47; Cl, 22.88; N, 9.04%. Found: C, 64.99; H, 3.73; Cl, 23.08; N, 9.29%.

*4-(4-Chloro-2-methyl-5-phenyl-7H-pyrrolo[2,3-d]pyrimidin-7-yl)-1,5-dimethyl-2-phenyl-1H-pyrazol-3(2H)-one* ***IVi***. Yield: 42%; M.P. 95-100°C; ^1^H NMR (DMSO-*d*_6_, 300 MHz) *δ* (ppm): 2.28–2.46 (s, 6H, 2∗CH_3_), 3.27 (s, 3H, N-CH_3_), 7.0–7.8 (m, 11H, Ar-H and C_6_-H); IR (KBr) *υ* (cm^−1^): 3070, 2850 (CH), 1700 (C=O), 1600 (C=C), 1570 (C=N); MS (EI)* m/z:* 429 (M^+^, 16%), 431 (M^+^ + 2, 4.7%); Anal. Calcd for C_24_H_20_ClN_5_O (429.90): C, 67.05; H, 4.69; Cl, 8.25; N, 16.29; O, 3.72%. Found: C, 67.42; H, 5.08; Cl, 8.57; N, 16.65; O, 4.02%.

#### 2.1.14. General Procedure for the Preparation of 4-Hydrazinopyrrolopyrimidines ** Vd–i** (Scheme 2)

*Method A.* Compound** IV (**0.01 mol) and hydrazine hydrate (8 mL, 0.015 mol, 98%) were refluxed in dry ethanol (30 mL) for 12 h. The solvent was removed under reduced pressure and the residues were recrystallized from methanol to give the target compounds** V**.

*Method B.* Compounds** Ii**,** j** (0.01 mol) in dry toluene (20 mL) and hydrazine hydrate (5 mL, 0.015 mol, 98%) were added with stirring at room temperature for 14 h. The solvent was removed under reduced pressure, and the residue was recrystallized from methanol to give** Vd**,** e**; Compounds** Vd**,** e** prepared by this method are identical in all respects (physical and spectral data) to that prepared from Method A.

*7-(3,4-Dichlorophenyl)-4-hydrazinyl-5,6-diphenyl-7H-pyrrolo[2,3-d]pyrimidine* ***Vd***. Yield: (A; 68%, B; 56%); M.P. 122–126°C; ^1^H NMR (DMSO-*d*_6_, 300 MHz) *δ* (ppm): 4.9–5.2 (brs, 2H, NH_2_, D_2_O exchangeable), 7.1–7.8 (m, 14H, Ar-H and NH, D_2_O exchangeable), 8.3 (s, 1H, C_2_-H); ^13^C NMR (DMSO-*d*_6_): *δ*117.45, 118.69, 126.21, 127.47, 128.4, 129.74, 130.2, 132.39, 132.64, 133.76, 134.84, 138.27, 141.05, 153.2, 153.8, 168.71 ppm; IR (KBr) *υ* (cm^−1^): 3420, 3350 (NH_2_) 3210 (NH), 1610 (C=C), 1580 (C=N); MS (EI)* m/z:* 446 (M^+^, 23%), 448 (M^+^ + 2, 14%), 450 (M^+^ + 4, 3.4%); Anal. Calcd for C_24_H_17_Cl_2_N_5_ (446.33): C, 64.58; H, 3.84; Cl, 15.89; N, 15.69%. Found: C, 64.95; H, 4.20; Cl, 16.23; N, 16.04%.

*4-(4-Hydrazinyl-5-phenyl-7H-pyrrolo[2,3-d]pyrimidin-7-yl)-1,5-dimethyl-2-phenyl-1H-pyrazol-3(2H)-one* ***Ve***. Yield: (A) 71%, (B) 60%; M.P. 142–146°C; ^1^H NMR (DMSO-*d*_6_, 300 MHz) *δ* (ppm): 2.42 (s, 3H, CH_3_), 3.13 (s, 3H, N-CH_3_), 4.8–5.1 (brs, 2H, NH_2_, D_2_O exchangeable), 7.1–7.8 (m, 11H, Ar-H, C_6_-H and NH, D_2_O exchangeable), 8.2 (s, 1H, C_2_-H); IR (KBr) *υ* (cm^−1^): 3440, 3330 (NH_2_) 3260 (NH), 1705 (C=O), 1600 (C=C), 1580 (C=N); MS (EI)* m/z:* 411 (M^+^, 28%), 412 (M^+^ + 1, 6.5%); Anal. Calcd for C_23_H_21_N_7_O (411.46): C, 67.14; H, 5.14; N, 23.83; O, 3.89%. Found: C, 67.52; H, 5.48; N, 24.15; O, 4.52%.

*7-Benzyl-4-hydrazinyl-2-methyl-5,6-diphenyl-7H-pyrrolo[2,3-d] pyrimidine* ***Vf***. Yield: 67%; M.P. 147–152°C; ^1^H NMR (DMSO-*d*_6_, 300 MHz) *δ* (ppm): 2.29 (s, 3H, CH_3_), 4.9–5.2 (brs, 2H, NH_2_, D_2_O exchangeable), 5.78 (s, 2H, Ph-CH_2_), 7.23–7.8 (m, 16H, Ar-H and NH, D_2_O exchangeable); IR (KBr) *υ* (cm^−1^): 3430, 3350 (NH_2_) 3250 (NH), 1605 (C=C), 1570 (C=N); MS (EI)* m/z:* 405 (M^+^, 29%), 406 (M^+^ + 1, 4.1%); Anal. Calcd for C_26_H_23_N_5_ (405.49): C, 77.01; H, 5.72; N, 17.27%. Found: C, 77.38; H, 6.05; N, 17.62%.

*4-(4-Hydrazinyl-2-methyl-5,6-diphenyl-7H-pyrrolo[2,3-d]pyrimidin-7-yl)-1,5-dimethyl-2-phenyl-1H-pyrazol-3(2H)-one* ***Vg***. Yield: 68%; M.P. 185–188°C; ^1^H NMR (DMSO-*d*_6_, 300 MHz) *δ* (ppm): 2.22–2.46 (s, 6H, 2∗CH_3_), 3.11 (s, 3H, N-CH_3_), 4.34–4.8 (brs, 2H, NH_2_, D_2_O exchangeable), 7.2–7.8 (m, 16H, Ar-H and NH, D_2_O exchangeable): IR (KBr) *υ* (cm^−1^): 3420, 3350 (NH_2_) 3240 (NH), 1700 (C=O), 1605 (C=C), 1560 (C=N); MS (EI)* m/z:* 501 (M^+^, 31%), 502 (M^+^+ 1, 5.4%); Anal. Calcd for C_30_H_27_N_7_O (501.58): C, 71.84; H, 5.43; N, 19.55; O, 3.19%. Found: C, 72.20; H, 5.81; N, 19.92; O, 3.55%.

*7-(3,4-Dichlorophenyl)-4-hydrazinyl-2-methyl-5,6-diphenyl-7H-pyrrolo[2,3-d]pyrimidine* ***Vh***. Yield: 59%; M.P. 142–146 °C; ^1^H NMR (DMSO-*d*_6_, 300 MHz) *δ* (ppm): 2.25 (s, 3H, CH_3_), 4.5–4.9 (brs, 2H, NH_2_, D_2_O exchangeable), 7.3–7.8 (m, 14H, Ar-H, NH, D_2_O exchangeable); IR (KBr) *υ* (cm^−1^): 3440, 3360 (NH_2_) 3250 (NH), 1610 (C=C), 1560 (C=N); MS (EI)* m/z:* 460 (M^+^, 31%), 462 (M^+^ + 2, 18%), 464 (M^+^ + 4, 4.9%); Anal. Calcd for C_25_H_19_Cl_2_N_5_ (460.36): C, 65.22; H, 4.16; Cl, 15.40; N, 15.21%. Found: C, 65.57; H, 4.52; Cl, 15.78; N, 15.56%.

*4-(4-Hydrazinyl-2-methyl-5-phenyl-7H-pyrrolo[2,3-d]pyrimidin-7-yl)-1,5-dimethyl-2-phenyl-1H-pyrazol-3(2H)-one* ***Vi***. Yield: 69%; M.P. 148–150°C; ^1^H NMR (DMSO-*d*_6_, 300 MHz) *δ* (ppm): 2.23–2.44 (s, 6H, 2∗CH_3_), 3.13 (s, 3H, N-CH_3_), 4.4–4.78 (brs, 2H, NH_2_, D_2_O exchangeable), 7.2–7.8 (m, 12H, Ar-H, C_6_-H and NH, D_2_O exchangeable); IR (KBr) *υ* (cm^−1^): 3430, 3360 (NH_2_) 3250 (NH), 1705 (C=O), 1600 (C=C), 1580 (C=N); MS (EI)* m/z:* 501 (M^+^, 31%), 502 (M^+^ + 1, 5.4%); Anal. Calcd for C_24_H_23_N_7_O (425.49): C, 67.75; H, 5.45; N, 23.04; O, 3.76%. Found: C, 68.12; H, 5.76; N, 23.37; O, 4.11%.

#### 2.1.15. General Procedure for the Preparation of 4-Thienopyrrolopyrimidine** VI d–f** (Scheme 2)

Compound** III** (0.01 mol) and thiourea (1.2 g, 0.02 mol) were refluxed in dry ethanol (20 mL) for 14 h. The reaction mixture was evaporated under reduced pressure and the residues were recrystallized from methanol to give the target compounds** VI**.

*7-(3,4-Dichlorophenyl)-5,6-diphenyl-3H-pyrrolo[2,3-d]pyrimidine-4(7H)-thione* ***VId***. Yield: 66%; M.P. 142–166°C; ^1^H NMR (DMSO-*d*_6_, 300 MHz) *δ* (ppm): 7.3–7.8 (m, 13H, Ar-H), 9.02 (s, 1H, C_2_-H), 11.71 (s, 1H, NH, D_2_O exchangeable); IR (KBr) *υ* (cm^−1^): 3250 (NH), 1630 (NH–C=S), 1560 (C=N); MS (EI)* m/z:* 447 (M^+^, 28%), 449 (M^+^ + 2, 18%), 451 (M^+^+ 4, 0.98%); Anal. Calcd for C_24_H_15_Cl_2_N_3_S (447.04): C, 64.29; H, 3.37; Cl, 15.81; N, 9.37; S, 7.15%. Found: C, 64.64; H, 3.74; Cl, 16.14; N, 9.73; S, 7.52%.

*1,5-Dimethyl-2-phenyl-4-(5-phenyl-4-thioxo-3H-pyrrolo[2,3-d]pyrimidin-7(4H)-yl)-1H-pyrazol-3(2H)-one* ***VIe***. Yield: 61%; M.P. 142–166°C; ^1^H NMR (DMSO-*d*_6_, 300 MHz) *δ* (ppm): 2.44 (s, 3H, CH_3_), 3.13 (s, 3H, N-CH_3_), 7.2–7.8 (m, 11H, Ar-H, and C_6_-H) 9.12 (s, 1H, C_2_-H), 12.10 (s, 1H, NH, D_2_O exchangeable); IR (KBr) *υ* (cm^−1^): 3230 (NH), 1700 (C=O), 1610 (NH–C=S), 1550 (C=N); MS (EI)* m/z:* 413 (M^+^, 30%), 414 (M^+^ + 1, 8.4%); Anal. Calcd for C_23_H_19_N_5_OS (413.49): C, 66.81; H, 4.63; N, 16.94; O, 3.87; S, 7.75%. Found: C, 67.16; H, 4.98; N, 17.31; O, 4.24; S, 8.12%.

*7-Benzyl-2-methyl-5,6-diphenyl-3H-pyrrolo[2,3-d]pyrimidine-4(7H)-thione* ***VIf***. Yield: 47%; M.P. 165–167°C; ^1^H NMR (DMSO-*d*_6_, 300 MHz) *δ* (ppm): 2.29 (s, 3H, CH_3_), 5.78 (s, 2H, Ph-CH_2_), 7.23–7.8 (m, 16H, Ar-H and NH, D_2_O exchangeable); IR (KBr) *υ* (cm^−1^): 3250 (NH), 1605 (NH–C=S), 1570 (C=N); MS (EI)* m/z:* 407 (M^+^, 26%), 408 (M^+^+ 1, 3.91%), 409 (M^+^+ 2, 0.81%); Anal. Calcd for C_26_H_21_N_3_S (407.53): C, 76.63; H, 5.19; N, 10.31; S, 7.87%. Found: C, 76.56; H, 5.11; N, 10.26; S, 7.80%.

#### 2.1.16. General Procedure for the Preparation of Substituted Carbonohydrazonoyl Derivatives** VII** (Scheme 3)

A mixture of** I** (0.01 mol) in concentrated HCl (10 ml) was cooled with stirring to 0–5°C under ice, and cooled sodium nitrite solution (2.5 g in 10 mL of water) was added to it dropwise during 30 minutes. The reaction mixture was then stirred for 30 minutes. Without separation, an ice-cold mixture of active methylene compounds (malononitrile and/or ethyl cyanoacetate) (0.015 mol) and sodium acetate (4.10 g; 0.05 mole) in ethanol (50 mL) were added dropwise with stirring for 15 min. The stirring was continued for 30 minutes under ice and the reaction mixture was then left for 12 h at room temperature. The precipitate was filtered off and recrystallized from ethanol/H_2_O to give** VII**.

*(3-Cyano-1-(3,4-dichlorophenyl)-4,5-diphenyl-1H-pyrrol-2-yl)carbon-hydrazonoyl dicyanide* ***VIId***. Yield: 56%; M.P. 102–106°C; ^1^H NMR (DMSO-*d*_6_, 300 MHz) *δ* (ppm): 6.71 (s, 1H, NH, hydrazone, D_2_O exchangeable), 7.3–7.8 (m, 13H, Ar-H); IR (KBr) *υ* (cm^−1^): 3290 (NH), 2320 (C*≡*N), 1695 (C=O), 1585 (C=N); MS (EI)* m/z:* 481 (M^+^, 19%), 482 (M^+^ + 2, 12.8%), 483 (M^+^ + 4, 2.3%); Anal. Calcd for C_26_H_14_Cl_2_N_6_ (481.33): C, 64.88; H, 2.93; Cl, 14.73; N, 17.46%. Found: C, 64.67; H, 2.78; Cl, 14.51; N, 17.12%.

*(3-Cyano-1-(1,5-dimethyl-3-oxo-2-phenyl-2,3-dihydro-1H-pyrazol-4-yl)-4-phenyl-1H-pyrrol-2-yl)carbonohydrazonoyl dicyanide* ***VIIe***. Yield: 51%; M.P. 110–114°C; ^1^H NMR (DMSO-*d*_6_, 300 MHz) *δ* (ppm): 2.42 (s, 3H, CH_3_), 3.11 (s, 3H, N-CH_3_), 6.71 (s, 1H, NH, hydrazone, D_2_O exchangeable), 7.2–7.8 (m, 11H, Ar-H, and C_6_-H); IR (KBr) *υ* (cm^−1^): 3290 (NH), 2320 (C*≡*N), 1695 (C=O), 1585 (C=N); MS (EI)* m/z:* 446 (M^+^, 17.8%), 447 (M^+^ + 1, 8.21%); Anal. Calcd for C_25_H_18_N_8_O (446.46): C, 67.25; H, 4.06; N, 25.10; O, 3.58%. Found: C, 67.54; H, 4.12; N, 25.23; O, 3.69%.

*Ethyl 2-(2-(1-Benzyl-3-cyano-4,5-diphenyl-1H-pyrrol-2-yl)hydrazono)-2-cyano-acetate* ***VIIf***. Yield: 48%; M.P. 125–130°C; ^1^H NMR (DMSO-*d*_6_, 300 MHz) *δ* (ppm): 1.31 (t, 3H, J = 6.8, CH_2_-CH_3_∗), 4.4 (q, 2H, J = 6.8, O-CH_2_), 5.62 (s, 2H, Ph-CH_2_), 6.8 (s, 1H, NH, hydrazone, D_2_O exchangeable), 7.2–7.8 (m, 15H, Ar-H, and C_6_-H); IR (KBr) *υ* (cm^−1^): 3290 (NH), 2320 (C*≡*N), 1695 (C=O), 1585 (C=N); MS (EI)* m/z:* 473 (M^+^, 18%), 474 (M^+^ + 1, 5.1%); Anal. Calcd for C_29_H_23_N_5_O_2_ (473.53): C, 73.56; H, 4.90; N, 14.79; O, 6.76%. Found: C, 73.48; H, 4.64; N, 14.63; O, 6.70%.

#### 2.1.17. General Procedure for the Preparation of Pyrazolyl Derivatives** VIII** (Scheme 3)

A mixture of compound** VII** (0.01 mol) and hydrazine hydrate (0.64 ml, 0.02 mole) in ethanol (30 mL) were heated under reflux for 8 h controlled by TLC. The solvent was concentrated and the reaction product was allowed to cool then pour on acidified ice/H_2_O. The product was filtered off, washed with water, dried, and recrystallized from ethanol to give** VIII**.

*2-(2-(3,5-Diamino-4H-pyrazol-4-ylidene)hydrazinyl)-1-(3,4-dichloro phenyl)-4,5-diphenyl-1H-pyrrole-3-carbonitrile* ***VIIId***. Yield: 55%; M.P. 120–124°C; ^1^H NMR (DMSO-*d*_6_, 300 MHz) *δ* (ppm): 6.5 (s, 4H, 2∗NH_2_, D_2_O exchangeable), 7.2–8 (m, 14H, Ar-H and NH, hydrazone, D_2_O exchangeable); IR (KBr) *υ* (cm^−1^): 3350–3280 (broad NH and NH_2_), 2320 (C*≡*N), 1695 (C=O), 1585 (C=N); MS (EI)* m/z:* 513 (M^+^, 12%), 515 (M^+^ + 2, 7.8%), 516 (M^+^ + 4, 2.4%); Anal. Calcd for C_26_H_17_Cl_2_N_7_O (513.09): C, 60.71; H, 3.33; Cl, 13.79; N, 19.06; O, 3.11%. Found: C, 60.98; H, 3.41; Cl, 13.95; N, 19.43; O, 3.27%.

*2-(2-(3,5-Diamino-4H-pyrazol-4-ylidene)hydrazinyl)-1-(1,5-dimethyl-3-oxo-2-phenyl-2,3-dihydro-1H-pyrazol-4-yl)-4-phenyl-1H-pyrrole-3-carbonitrile* ***VIIIe***. Yield: 61%; M.P. 135–138°C; ^1^H NMR (DMSO-*d*_6_, 300 MHz) *δ* (ppm): 2.42 (s, 3H, CH_3_), 3.11 (s, 3H, N-CH_3_), 6.48 (s, 4H, 2∗NH_2_, D_2_O exchangeable), 6.89 (s, 1H, NH, hydrazone, D_2_O exchangeable), 7.3–8 (m, 11H, Ar-H, and C_6_-H); IR (KBr) *υ* (cm^−1^): 3340–3290 (broad NH and NH_2_), 2320 (C*≡*N), 1695 (C=O), 1585 (C=N); MS (EI)* m/z:* 478 (M^+^, 15.2%), 479 (M^+^ + 1, 4.46%); Anal. Calcd for C_25_H_22_N_10_O (478.51): C, 62.75; H, 4.63; N, 29.27; O, 3.34%. Found: C, 62.64; H, 4.47; N, 29.02; O, 3.09%.

*2-(2-(3-Amino-5-hydroxy-4H-pyrazol-4-ylidene)hydrazinyl)-1-benzyl-4,5-diphenyl-1H-pyrrole-3-carbonitrile* ***VIIIf***. Yield: 56%; M.P. 97–100°C; ^1^H NMR (DMSO-*d*_6_, 300 MHz) *δ* (ppm): 5.61 (s, 2H, CH_2_), 6.45 (s, 4H, 2∗NH_2_, D_2_O exchangeable), 6.8 (s, 1H, NH, hydrazone, D_2_O exchangeable), 7.2–7.9 (m, 15H, Ar-H).; IR (KBr) *υ* (cm^−1^): 3340–3270 (broad NH and NH_2_), 2310 (C*≡*N), 1690 (C=O), 1575 (C=N); MS (EI)* m/z:* 459 (M^+^, 10%), 460 (M^+^ + 1, 2.91%); Anal. Calcd for C_27_H_21_N_7_O (459.50): C, 70.57; H, 4.61; N, 21.34; O, 3.48%. Found: C, 70.65; H, 4.69; N, 21.07; O, 3.41%.

## 3. Biological Screening 

### 3.1. Animals

The complete course of the experiment was conducted using male Wistar albino rats (200–250 g), reared and maintained in the animal house of the institution and provided free access to pelleted food and water* ad libitum*. The rats were maintained in a controlled environment (12 h light and dark cycle) for about a week for acclimatization. The protocol of the study was approved by the animal ethics committee of the Faculty of Pharmacy, Helwan University (10-01-2012). The study was conducted in accordance with the EC, directive 86/609/EEC for animal experiments.

### 3.2. Dose Determination

Glimepiride (Amaryl) was used as a standard antidiabetic (4 mg/kg) in 1% gum acacia and administered orally [32]. Equivalent doses of all derivatives were calculated according to their molecular weight [M*·*wt].

### 3.3. Sucrose-Loaded Model (SLM)

Male Wistar rats were fasted overnight. Blood was collected initially and then the compounds were given to corresponding groups consisting of six rats each by oral gavage. A sucrose load of (10 gm/kg) body weight was given to each rat after half an hour posttest treatment. Blood was collected in 30, 60, 90, and 120 min after sucrose load [33]. The percentages (%) fallen in blood glucose level were calculated according to the AUC method.

### 3.4. Toxicity Study

The derivatives, which showed antihyperglycemic activity in this study, were subjected to* in vivo* acute toxicity study by testing their effect on serum liver and kidney markers.

### 3.5. Induction of Experimental Diabetes

Diabetes was induced in overnight fasted rats with a single intraperitoneal injection of streptozotocin (STZ) (Sigma-Aldrich, Co., St. Louis, USA. Catalog number: 1001062761) in a dose of 65 mg/kg. STZ was freshly dissolved in ice cold citrate buffer (0.01 M, pH 4.5) prior to injection [34]. After 48 h, rats showing blood glucose level  ≥ 200 mg/dl were included in the experiment [35].

### 3.6. Experimental Design

Seventy-six rats (fourteen groups of 5-6 rats each) were used to investigate the antihyperglycemic effect of 12 pyrrole and pyrrolo pyrimidine derivatives.* Group *1 was diabetic control;* Group *2, diabetic + Glimepiride (Amaryl) (4 mg/kg), served as a reference antidiabetic drug.* Groups *(3–14) were given the various pyrrole derivatives (**Ia–e**,** IVg**,** VIf**,** VIIa**,** b**,** f**, and** VIIIf**,** a**, resp.). The treated groups administered the Amaryl and different derivatives orally.

### 3.7. Methodology

For each group, blood glucose was estimated at zero, one, two, four, and six hours after oral administration of derivatives using glucometer (Gluco Dr Super Sensor, AllMedicus Co., Ltd., Anyang, Gyeonggi, Korea).

Alanine aminotransferase (ALT) and aspartate aminotransferase (AST) activities in serum were measured according to the Reitman-Frankel calorimetric transaminase procedure [36], whereas alkaline phosphatase (ALP) was assayed by the kinetic enzymatic method by measuring the rate of hydrolysis of p-nitrophenyl phosphate by ALP according to Henry [37]; all were measured as indicators of hepatic injury. Serum creatinine levels were assayed as an indicator for renal injury in the samples by a calorimetric method [38], using commercial diagnostic kits (Diamond Diagnostics, Egypt).

### 3.8. Statistical Analysis

Data were represented as mean area under curve (AUC) ± SD. Significant differences between groups was tested using GraphPad InStat (Graph software Inc., V 3.05, Ralph Stahlman, Purdue University). Appropriate graphs were plotted using Microsoft Excel 2007. *P* value less than 0.05 was considered statistically significant.

## 4. Results and Discussion

### 4.1. Chemistry

The target pyrrole* o*-amino carbonitriles** Ia**,** b**, and** d** were prepared by the reaction [26–31] of benzoin with appropriate amines and malononitrile in nonpolar solvent. On the other hand,** Ic** and** e** were obtained by condensation of *α*-(arylamino)-acetophenone with malononitrile in sodium ethoxide/ethanol.

Compounds** Ia–e** were utilized for the preparation of pyrrole derivatives** If–o** using appropriate reagents and reaction conditions; heating** Ia–e** with triethyl orthoformate (TEOF) afforded the corresponding 2-ethoxy methylamino derivative** If–j**, while, on react with acetic anhydride, the corresponding 2-acetylamino** Ik–o** were afforded, as revealed in Scheme 1.

On the other hand, the pyrrole derivatives** Ia–e** were converted to the corresponding pyrrolo[2,3-*d*]pyrimidine-4-ones** IIa–j** via condensation with formic acid [39, 40] and/or AcOH/HCl [28, 41], as revealed in Scheme 2.

Interaction [41] of** Ia–e** with formamide afforded the corresponding 4-amino pyrrolo[2,3-*d*]pyrimidines** IIIa–e**, which can also be prepared via stirring of the imidate** I f–j** with ammonium hydroxide at room temperature, as revealed in Scheme 2. The reaction of pyrrole* o*-amino carbonitriles** Ia–e** with thiourea in ethanol was reported [42] to afford the corresponding 4-aminopyrimidine-2-thione** IIIf–j**.

Pyrrolopyrimidinones** IIa–j** were converted [41, 43, 44] to its corresponding 4-chloro derivative** IVa–j** by refluxing with phosphorus oxychloride, as revealed in Scheme 2.

The 4-chloro** IVa–j** were utilized for the preparation of pyrrolopyrimidine derivatives** Va–j** and** VIa–j** using appropriate reagents and reaction conditions [40, 44]: the synthesis of certain 4-hydrazino-*7H*-pyrrolo[2,3-*d*]pyrimidines** Va–j** by hydrazinolysis of the corresponding 4-chloro analogues. Yet, when 4-chloro analogues** IVa–j** and thiourea were heated [45] in absolute ethanol, the pyrrolopyrimidine-4(*3H*)-thiones** VIa–f** were obtained, as revealed in Scheme 2. Diazotisation reaction of amino group in 2-amino-pyrrole, followed by coupling of the diazonium salt with active methylenes (ex: malononitrile) has been reported [46–48].

Diazotization of** Ia–e** using a mixture of sodium nitrite and HCl (without acetic acid) at 0–5°C, without separation, adding an active methylene compounds, namely, malononitrile and/or ethyl cyanoacetate in ethanol in the presence of sodium acetate afforded the corresponding hydrazono derivatives** VIIa–i**. This reaction could be explained via formation of the diazonium chlorides at first, which in addition to malononitrile afforded** VIIa–i**. Cyclization of hydrazono derivatives 2 using hydrazine hydrate in boiling ethanol leads to the formation of the corresponding pyrazolin-5-one derivatives** VIIIa–i**, as revealed in Scheme 3.

### 4.2. Biological Activities

Twelve of the synthesized Pyrroles and pyrrolopyrimidines were evaluated for their antihyperglycemic activity using both streptozotocin models of diabetes and sucrose load model [32–35]. The synthesized compounds were assessed for their antihyperglycemic activity, which is comparable to Glimepiride (Amaryl) the standard antihyperglycemic drug, by comparing the mean area under the curve (AUC) for the blood glucose level between the different studied groups. The proved pyrrole derivatives, which showed promising decrease in the serum blood glucose level, were subjected to test their toxicity* in vivo* on serum liver and kidney markers.

The tested compounds were classified into 2 main groups: first, the open form pyrrole derivatives, namely,** Ia–e (**pyrrole* o*-amino carbonitriles), hydrazone derivatives** VIIa**,** b**, and** f**, and pyrazolin-5-one derivatives** VIIIa**,** f**;second, the pyrrolopyrimidines, namely, 4-chloro** IVg** and 4-thio derivatives** VIf.**

Only the open form pyrrole derivatives, namely,** I a**,** c**, and** e** (pyrrole* o*-amino carbonitriles), induced a significant decrease in blood glucose level in the sucrose load model (17.4%, 18%, and 16.7%, respectively) compared to the untreated normal control. Moreover, they induced significant decrease in blood glucose level in the STZ model of diabetes (33.3%, 35.3%, and 29.5%, respectively) compared to the diabetic control group, as depicted in Table 1.

**Figure 3:**
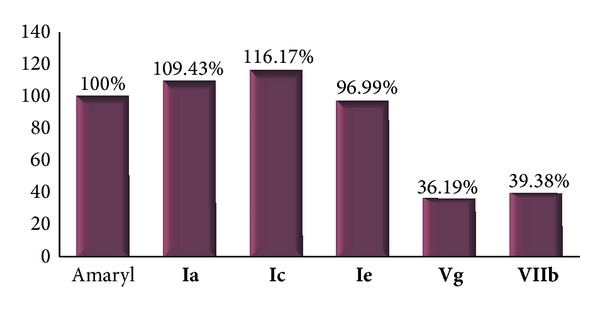
Potency of antihyperglycemic derivatives compared to Amaryl.

Comparing the antihyperglycemic activity of the these compounds with that of the reference antidiabetic drug (Amaryl) showed that compounds** Ia**,** Ic**, and** Ie** showed significant decrease in the blood glucose level (109.4%, 116.2%, and 97%, respectively) when compared to the activity of Amaryl, as shown in Figure 3.

Among the pyrrolopyrimidines, only the 4-chloro** IVg** (also bearing the antipyrine moiety at N-pyrrole) showed marked but not significant decrease in blood glucose level 11.2% compared to the diabetic control group, as shown in Table 1.

Studying the acute toxicity of the promising antihyperglycemic derivatives** Ia**,** c**, and** e** on the rats showed that the levels of sera ALT, AST, ALP, and creatinine were not significantly changed from that of the control untreated group and, also, the rats did not die or show any toxicity symptoms, as shown in Table 2.

**Figure 4:**
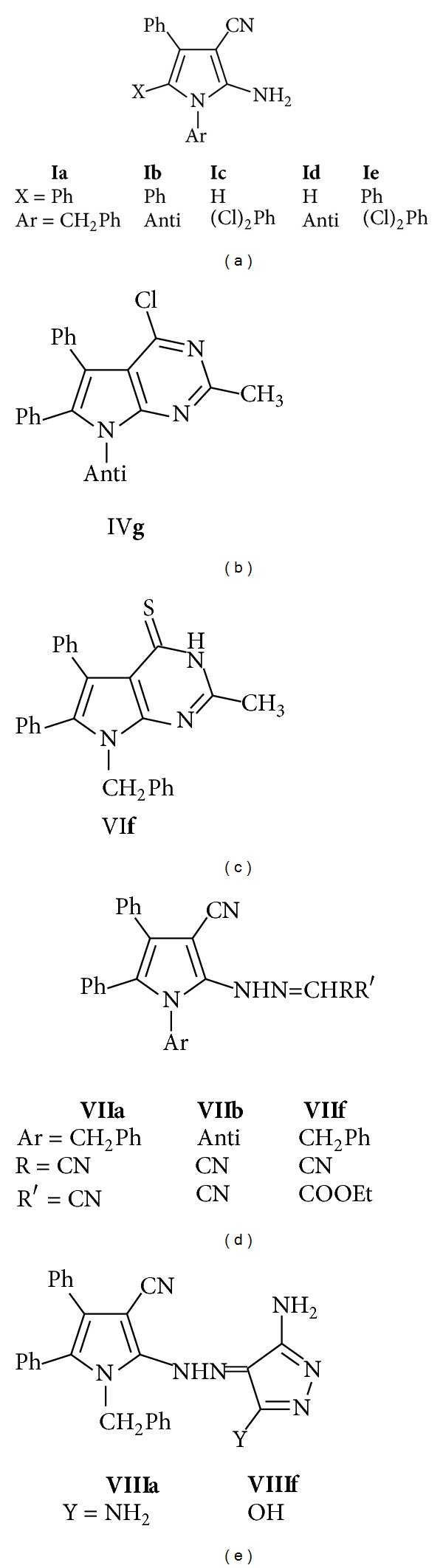
Pyrrole and Pyrrolopyrimidines derivatives; evaluated as antihyperglycemic agents.

To analyze structure-activity relationships, three structural components were considered: the nature of the heterocycle nucleus, the nature of the side chain of the heterocycle system, and the function of the side chain, as shown in Figure 4.

First, the influence of the nature of the heterocyclic system was easily observed as pyrrole (**I a**,** c**, and** e**) derivatives have show superior activity over pyrrolopyrimidines** IVg** and** VIf**.

Regarding the side chain function, for the pyrroles derivatives, the free amino group in pyrrole* o*-amino carbonitriles** I a**,** c**, and** e** conferred the greater activity over the hydrazone derivatives** VIIa**,** b**, and** f** which showed a marked activity over the pyrazolin-5-one derivatives** VIIIa**,** f**, which have no activity. For the pyrrolopyrimidines, the 4-chloro** IVg** confers markedly but not significantly higher activity than the 4-thio derivatives** VIf**.

Finally, the influence of the nature of the side chain on the heterocycle system, among the active compounds the antipyrine bearing* N7-pyrrole* (**I e** and** IVg**) showing a good activity over the benzyl (**VIf**,** VIIa** and** VIIIa**).

## 5. Conclusion

In the present study, we described a straightforward and efficient synthesis of some pyrroles and pyrrolo[2,3-*d*]pyrimidine and also,we examined their effects as antihperglycemic agents. The structure-activity relationship (SAR) results indicated that the pyrroles** Ia**,** c**, and** e** containing amino and cyano groups displayed good to moderate antihyperglycemic activity profile compared to control. On diazotization of the amino group in** VII** and** VIII**, this did not enhance the activity. The introduction of chloro group to** IVg** resulted in an enhanced antihyperglycemic activity of the pyrrolopyrimidine analogs over the hydrazine derivatives. These results and others demonstrated that the synthesized pyrrole and pyrrolopyrimidine compounds are promising antihyperglycemic agents.

## Figures and Tables

**Table 1:** Effect of various treatments on the mean area under curve (AUC) of blood glucose levels in rats.

Tested compound(s)	% reduction in blood glucose compared to control	
	SLM	STZ
Amaryl [Standard drug]	**27.7** ^ a^	**30.4** ^ a^
[**Ia**]	**17.4** ^ a^	**33.3** ^ a^
[**Ib**]	**10.9** ^ a^	NA
[**Ic**]	**18** ^ a^	**35.3** ^ a^
[**Id**]	NA	NA
[**Ie**]	**16.7** ^ a^	**29.5** ^ a^
[**IVg**]	**13** ^ a^	**11.2** ^ a^
[**VIf**]	NA	NA
[**VIIa**]	NA	NA
[**VIIb**]	NA	NA
[**VIIf**]	NA	NA
[**VIIIa**]	NA	NA
[**VIIIf**]	NA	NA

NA = not active.

^a^Considered significant compared to control (*P* ≤ 0.05).

SLM: Sucrose-Loaded Model; STZ: Streptozotocin model of diabetes.

**Table 2:** Effect of compounds **Ia**, **Ic,** and **Ie** on ALT, AST, ALP, and creatinine.

Parameter	Control	**Ia**	**Ic**	**Ie**
ALT (U/L)	22.6 ± 4.6	17.9 ± 2.7	27.16 ± 6.3	20.5 ± 3.1
AST (U/L)	63.4 ± 14.6	69.4 ± 9.5	72 ± 7.8	62.7 ± 9.7
ALP (U/L)	70.6 ± 15	68.5 ± 12.3	63.8 ± 15.4	73.9 ± 13.2
Creatinine (mg/dL)	0.83 ± 0.13	0.8 ± 0.16	0.63 ± 0.09	0.77 ± 0.14
